# Molecular mechanism and potential role of mitophagy in breast cancer

**DOI:** 10.7150/jca.140132

**Published:** 2026-09-24

**Authors:** Xin Yang, Youqin Zeng, Ling Zhang, Yuting Ye, Siyu Chen, Changyou Wei, Ping Leng

**Affiliations:** 1 College of Medical Technology, Chengdu University of Traditional Chinese Medicine, China.; 2 Chongqing Key Laboratory of Sichuan-Chongqing Co-construction for Diagnosis and Treatment of Infectious Diseases Integrated Traditional Chinese and Western Medicine, China.; 3 Department of Clinical Laboratory, Pengzhou Hospital of Traditional Chinese Medicine, China.; 4 School of Public Health, Chengdu Medical College, China.

**Keywords:** mitophagy, breast cancer, cell death, therapy

## Abstract

Breast cancer remains the most commonly diagnosed malignancy among women worldwide and a leading cause of cancer related mortality in this population. Mitochondrial dysfunction is increasingly recognized as a hallmark of breast cancer, contributing to tumor initiation, progression, and therapeutic resistance. Mitophagy, a selective form of autophagy that eliminates damaged or dysfunctional mitochondria, is essential for maintaining cellular and mitochondrial homeostasis. Accumulating evidence indicates that mitophagy plays a context dependent role in breast cancer. Impaired mitophagy permits the accumulation of damaged mitochondria and promotes tumor growth, migration, and invasion, whereas appropriately activated mitophagy removes damaged mitochondria and suppresses tumor progression. This review synthesizes the molecular mechanisms of mitophagy, its crosstalk with ferroptosis, and its context dependent roles in breast cancer development, with the aim of offering therapeutic insights that may inform breast cancer treatment.

## 1. Introduction

Breast cancer exhibits a rising global incidence 1. Despite advances in early detection and treatment, breast cancer remains the most commonly diagnosed malignancy in women globally and the second leading cause of cancer-related death, accounting for approximately 2.3 million new cases and 670,000 deaths in 2022 2. The advent of novel breast cancer therapies has been tempered by the occurrence of adverse effects 3, notably organ damage, drug resistance, and an elevated risk of disease recurrence 4-6. Therefore, identifying effective therapeutic targets and developing novel treatment strategies remain a current priority.

Regulated cell death (RCD) refers to a type of cell death that is controlled by defined molecular pathways 7. Cell death may occur through genetically programmed suicide mechanisms such as apoptosis, or be triggered by dysregulated metabolic processes such as ferroptosis 8. Various cell death modalities play significant roles in regulating tumor development, progression and drug resistance through different signaling pathways. Autophagy is a cellular process that promotes the degradation of intracellular macromolecules or damaged organelles 9. Autophagy can be subdivided into nonselective forms (e.g., microautophagy and macroautophagy) or selective forms (including mitophagy, endoplasmic reticulum autophagy (ER-autophagy), and ribosomal autophagy) 10,11. By identifying and eliminating damaged or excess mitochondria, the selective autophagic process known as mitophagy regulates mitochondrial number and function to maintain cellular balance 12. Accumulating evidence implicates mitophagy in cancer pathogenesis, particularly in breast cancer 13. Mitochondrial dysfunction underpins key aspects of tumorigenesis, including cancer cell survival and drug resistance 14,15. Due to a dual role and dynamic participation in the inhibitory and promoting processes of tumors, the mechanism of mitophagy in the occurrence and development of breast cancer is rather complex. Furthermore, the therapeutic potential of targeting this process remains to be fully elucidated 16. Therefore, by investigating the role and molecular processes of mitophagy in breast cancer progression, this review aims to summarize the role and molecular mechanisms of mitophagy in breast cancer progression and to highlight potential therapeutic strategies.

## 2. Two Pathways of Mitophagy: Ubiquitin-dependent Pathways and Ubiquitin-independent Pathways

### 2.1 Ubiquitin-dependent pathways

The PTEN-induced putative kinase 1-Parkin RBR E3 ubiquitin-protein ligase (PINK1-Parkin) pathway is the most extensively studied mechanism of mitophagy 17. The N-terminus of PINK1 contains the mitochondrial outer membrane localization signal, which is responsible for maintaining the localization of PINK1 during depolarization of the outer mitochondrial membrane (OMM). The C-terminus of PINK1 is involved in the regulation of its kinase activity 18. Under conditions of mitochondrial damage or depolarization, PINK1 import into the inner mitochondrial membrane is impaired, leading to its accumulation on the OMM 14. There, PINK1 phosphorylates ubiquitin at Ser65 to generate phospho Ser65 ubiquitin and subsequently phosphorylates Parkin at Ser65 within its Ubl domain 19-21. Phospho Ser65 ubiquitin acts as both an allosteric activator and a membrane receptor for Parkin, accelerating UBCH7 ubiquitin thioester discharge, relieving autoinhibition of the catalytic cysteine, and recruiting Parkin to depolarized mitochondria to establish a feed forward amplification loop 20-22. Binding of phospho Ser65 ubiquitin to Parkin also induces a conformational change that enhances Parkin Ser65 accessibility, promoting optimal phosphorylation by PINK1 23. This dual phosphorylation mechanism ultimately triggers autophagic lysosomal degradation of damaged mitochondria. In addition to Parkin, several ubiquitin E3 ligases such as SMURF1 24, Gp78 25, SIAH1 26, and ARIH1 27 are involved in the ubiquitination of mitochondrial proteins and the induction of mitophagy 28-30. In contrast, mitochondrial E3 ubiquitin protein ligase 1 (MUL1) restrains Parkin-mediated mitophagy by maintaining ER-mitochondrial contacts 31. Upon Parkin activation, the E3 ubiquitin ligase conjugates polyubiquitin chains to outer mitochondrial membrane proteins, including voltage dependent anion channels (VDACs) and mitofusins (MFN1 and MFN2) 31. This ubiquitinated surface is recognized by cytosolic autophagy adaptors, such as optineurin (OPTN) 32, nuclear dot protein 52 kDa/CALCOCO2 (NDP52) 33, sequestosome 1 (SQSTM1/p62), Tax1 binding protein 1 (TAX1BP1) 34, and neighbor of BRCA1 gene 1 (NBR1) 35, each of which contains ubiquitin-binding domains (UBDs) that engage the polyubiquitin moieties on the mitochondrial outer membrane. These adaptors simultaneously use their LC3-interacting region (LIR) motifs to associate with autophagy-related protein 8 (ATG8) family proteins on the nascent autophagosomal membrane 36. Through this dual engagement, the adaptors serve as molecular bridges that couple the ubiquitinated mitochondrion to the autophagosome, facilitating encapsulation and subsequent lysosomal delivery of the damaged organelle for degradation. Table 1 provides a detailed comparison of the two modes of mitophagy.

### 2.2 Non-ubiquitin dependent pathways mediated by mitophagic receptors

Certain proteins on the mitochondrial surface can function directly as mitophagy receptors, enabling ubiquitin-independent mitophagy 37. Owing to their intrinsic LIR domains, these proteins can directly bind microtubule-associated protein 1A/1B-light chain 3 (LC3) and/or GABA receptor-associated protein (GABARAP), thereby initiating mitophagy independently of ubiquitination 38.

#### 2.2.1 The BNIP3/NIX receptor

The BCL2/adenovirus E1B 19-kDa interacting protein 3 (BNIP3) and BCL2 interacting protein 3 like (BNIP3L/NIX) receptor-mediated pathways are important and widely regulated under many physiological conditions. The single-channel C-terminal transmembrane (TM) structural domain of the transmembrane proteins BNIP3L/NIX, which are autophagy receptors, serves as an anchor for the proteins to the OMM 39. The majority of BNIP3 and BNIP3L/NIX are directed to the cytoplasm by the TM structural domain, where these receptors use their LIR and/or microtubule-binding domains to recruit the cellular autophagy machinery to the mitochondria 19. Phosphorylation of Ser17, which is adjacent to the BNIP3 LIR motif, is required for its binding to LC3B, whereas synergistic phosphorylation at both Ser17 and Ser24 enhances BNIP3's affinity for GABARAPL2 and subsequently augments mitophagy 40,41. Mitophagy induction leads to the dephosphorylation of BNIP3L/NIX, which facilitates its dimerization. The resulting dimer then undergoes dual phosphorylation on its LIR domain, a critical step for its full activation. Dimerization thereby enhances autophagosome recruitment and mitophagy efficiency 42. Previous studies have demonstrated that the SCFFBXL4 family localizes to the mitochondrial outer membrane of non-stressed cells and mediates the ubiquitination and degradation of the mitophagy receptors BNIP3 and BNIP3L/NIX to inhibit mitophagy 43. In addition, accumulating evidence has documented that JNK1/2 phosphorylates BNIP3 at the Ser60/Thr66 location during hypoxia, preventing BNIP3 from being degraded by proteases and encouraging mitophagy by enabling BNIP3 to directly bind to LC3 44.

#### 2.2.2 FUNDC1 receptor

FUN14 domain containing 1 (FUNDC1) is an OMM localized protein that binds to LC3 and promotes mitophagy under hypoxic conditions 45. High expression of FUNDC1 enhances mitophagy and cell proliferation, while low expression of FUNDC1 inhibits hypoxia-induced mitophagy and cell proliferation 46,47. The 155-amino-acid human FUNDC1 protein is composed of an N-terminal cytoplasmic domain, a C-terminal domain, and an intermembrane space segment 48. Mutations or deletions in the conserved LIR sequence of FUNDC1 inhibit its interaction with LC3 and subsequent mitophagy 49.

The structural features of FUNDC1 allow it to regulate mitophagy through phosphorylation and dephosphorylation in response to changes in intracellular signaling. FUNDC1 is normally stabilized on the OMM, and phosphorylation of Ser13 and Tyr18 by SRC kinase and casein kinase 2 (CK2) adversely influences the interaction between FUNDC1 and ATG8 proteins 50. The dephosphorylation of FUNDC1, together with the inactivation of Src and CK2, enables FUNDC1 to bind LC3 and trigger mitophagy under hypoxia 51,52. Figure 1 briefly summarizes the two pathways of mitophagy and the related molecules involved.

## 3. The Role of Mitophagy-Related Molecules in Breast Cancer

### 3.1 PINK1/Parkin in breast cancer

The mRNA expression of PINK1 is downregulated in most tumors. Despite the significant prognostic value of PINK1 expression in cancer, PINK1 plays a protective or deleterious role in different types of cancer 53. However, PINK1 exerts opposing effects on breast cancer cells depending on the cellular context. Mechanistically, PINK1 activation promotes Parkin-mediated ubiquitination and degradation of breast cancer susceptibility gene 1 (BRCA1), whereas PINK1 inhibition stabilizes BRCA1 expression 54. Elevated BRCA1 levels, in turn, correlate with enhanced proliferative activity in breast cancer cells, as demonstrated by both in vitro assays and clinical transcriptomic analyses linking high BRCA1 mRNA to increased proliferation scores 55. Consequently, in contexts wherein PINK1 inhibition gives rise to BRCA1 accumulation, PINK1 may exert a suppressive rather than promotive influence on breast cancer cell proliferation, thereby underscoring the context-dependent nature of its biological function. According to another study, PINK1 deficiency can inhibit breast cancer cells from growing 56. As a tumor suppressor, Parkin is a crucial protein in the regulation of mitophagy 57,58. Parkin expression is frequently dysregulated across diverse human malignancies. In breast cancer, compared with adjacent normal tissues, the mRNA and protein levels of Parkin are down-regulated 59. These findings emphasize the importance of mitophagy in the pathophysiology of breast cancer and the function of PINK1 and Parkin in controlling cellular metabolism.

### 3.2 FUNDC1 and BNIP3 in breast cancer

High levels of FUNDC1 enhance oxidative phosphorylation and facilitate the proliferation and growth of tumor cells 60. Compared with normal breast tissues, FUNDC1 expression is significantly upregulated in breast cancer tissues. Moreover, elevated FUNDC1 protein levels are associated with a more aggressive disease phenotype in breast cancer patients 61. Additionally, FUNDC1 is essential for encouraging breast cancer cells to proliferate, migrate, and invade 62. Furthermore, the overexpression of BNIP3 occurs in the early stage of breast cancer 63. Functionally, breast cancer cells with high expression of BNIP3 exhibit enhanced metabolism, proliferation, migration, and drug resistance, along with higher antioxidant capacity 64.

## 4. The Role of Mitophagy in the Development of Breast Cancer

The functional significance of mitophagy in breast cancer is context-dependent, governed by three interrelated variables: tumor subtype, metabolic phenotype, and the intensity of mitochondrial stress. Mitochondria generate adenosine triphosphate (ATP) primarily through oxidative phosphorylation, which is fueled by the tricarboxylic acid cycle (TCA). When damaged, ATP synthesis declines and reactive oxygen species (ROS) accumulate, prompting the selective removal of dysfunctional mitochondria through mitophagy to maintain cellular homeostasis 65. Mitophagy is triggered by hypoxia, nutrient deprivation, oxidative stress, and pharmacological agents, primarily through hypoxia-inducible factor 1 subunit alpha (HIF1α) and NF-κB signaling 66. The regulation and consequences of mitophagy in breast cancer vary considerably according to the molecular and metabolic context 67.

Subtype specificity and metabolic phenotype determine the baseline dependency on mitophagy. In triple negative breast cancer (TNBC), a subtype marked by high metabolic plasticity, mitophagy predominantly acts as a pro-survival mechanism. Divalent metal transporter 1 (DMT1) inhibition triggers PINK1/Parkin-dependent mitophagy and promotes pulmonary metastasis 68. Conversely, NLRX1 deficiency impairs proliferation and migration in oxidative phosphorylation-dependent TNBC cells, revealing their reliance on intact mitophagy 69. In luminal and HER2-positive subtypes, however, PINK1 inhibition increases BRCA1 expression and promotes proliferation, suggesting a tumor-suppressive role for mitophagy in these contexts 54,55. The PINK1-PGK2 axis connects mitophagy to glycolytic reprogramming, with PINK1 overexpression directly enhancing mitophagy and inducing a glycolytic phenotype that promotes breast cancer proliferation 14. FUNDC1 further illustrates this complexity: it activates the calcium-NFATC1-BMI1 axis to drive migration and proliferation, while also suppressing epithelial-mesenchymal transition via ROS reduction, a duality that underscores the context-dependent nature of mitophagy in breast cancer 62.

The intensity and duration of mitochondrial damage represent a second critical determinant. Mild, transient stress elicits a homeostatic response that restores mitochondrial integrity and supports cell survival. In contrast, sustained or hyperactivated mitophagy leads to excessive mitochondrial depletion and bioenergetic failure. This threshold effect is exemplified by Unc-51 like autophagy activating kinase 1 (ULK1) deficiency under hypoxia, which suppresses mitophagy, promotes dysfunctional mitochondrial accumulation, and enhances invasive potential and osteolytic bone metastasis 70. Similarly, HIF-1-induced MRPL52 activates PINK1/Parkin-dependent mitophagy under hypoxia, suppressing apoptosis and promoting metastasis via the ROS-Notch1-Snail axis 71. BRCA1 loss impedes stress-induced mitophagy by blocking ATM-AMPK-DRP1-dependent fission while activating the NLR family pyrin domain containing 3 (NLRP3) inflammasome, thereby creating a tumor-permissive microenvironment that favours metastasis 72. Thus, the same pathway that preserves cellular integrity under acute stress can, when chronically overstimulated, trigger cell death.

Metabolic phenotype further modulates this duality. Cells heavily reliant on oxidative phosphorylation (OXPHOS) are particularly vulnerable to mitophagy inhibition, which compromises mitochondrial function and viability. In contrast, glycolytic cells exhibit relative resistance, suggesting that the therapeutic window for mitophagy-targeted interventions is contingent upon the tumor's metabolic state, a stratification that may facilitate the identification of responsive tumors.

Recent studies have substantiated this paradigm by positioning mitophagy as a mechanistic hub linking metabolic reprogramming to therapeutic resistance in TNBC. Epigenetic reactivation of TNFRSF19 suppresses PINK1/Parkin-mediated mitophagy by disrupting TGFBR1-SMAD3 signaling, thereby sensitizing TNBC to doxorubicin 73. Lysyl oxidase (LOX) inhibition disrupts mitochondrial homeostasis via uncoupling glucose metabolism from Parkin-mediated mitophagy, thereby generating a targetable ferroptotic vulnerability 74. Conversely, TBX20 drives doxorubicin resistance through ABCC1 upregulation coupled with mitophagy suppression, illustrating the paradoxical pro-resistance outcome of mitophagy inhibition in certain settings 75. The tumor suppressor CIDEC impairs mitochondrial fitness and abrogates protective autophagy via cGMP/PKG pathway inhibition, exerting context-dependent antitumor effects 76. Furthermore, mitochondrial PDK1 potentiates PINK1/Parkin-dependent mitophagy under hypoxia, thereby fueling bevacizumab resistance and malignant progression, with its targeting restoring chemosensitivity 77. Collectively, these findings underscore that the therapeutic exploitation of mitophagy, whether via activation or inhibition, demands careful contextualization to tumor subtype, metabolic state, and the prevailing resistance mechanism.

## 5. Mitophagy and Ferroptosis in Breast Cancer

Ferroptosis is a recently identified kind of iron-dependent cell death that is different from apoptosis and other types of cell death. It is typically accompanied by severe lipid peroxidation and the accumulation of iron ions 78. Among the identified autophagic processes, ferroptosis is regulated by nuclear receptor coactivator 4 (NCOA4)-mediated ferritin autophagy 79, BECN1-mediated inhibition of system Xc⁻ 80, and PINK1 associated mitophagy 81. BNIP3 knockdown upregulates NCOA4 and promotes ferroptosis, suggesting a functional interplay between BNIP3-mediated mitophagy and NCOA4-mediated ferritinophagy in iron homeostasis and ferroptosis regulation 82. A schematic overview of the molecular crosstalk between mitophagy and cell death subroutines, including pyroptosis, apoptosis, and the core autophagy and mitophagy machinery, is presented in Figure 2.

Ferritin autophagy plays an important role in ferroptosis, and this process is further enhanced by interactions between mitophagy and the ferritinophagy pathway 83. Singh et al. attribute high glucose induced ferroptosis to the upregulation of thioredoxin-interacting protein, which promotes redox stress, ferritinophagy, mitochondrial damage, and lysosomal instability, thereby enabling the iron-dependent Fenton reaction and ∙OH generation 84. Moreover, ferroptosis in turn stimulates autophagy. O-linked β-N-acetylglucosamine (O-GlcNAc) transferase inactivation occurs concurrently with ferroptosis induction, resulting in ferritin de-O-GlcNAc and NCOA4-mediated activation of ferritin autophagy and mitophagy 85,86. Together, ferroptosis and ferritin autophagy provide an unstable iron substrate for the Fenton reaction, which accelerates the production of ROS and lipid peroxidation, both of which in turn trigger mitophagy 87.

Although mitophagy and ferroptosis are broadly interconnected through the feed forward loop described above, the net outcome of this interaction depends on which mitophagy receptor is engaged. Several mitochondrial molecules regulate ferroptosis through their effects on mitophagy, and the direction of regulation varies with the receptor and cellular context. For example, BNIP3- and BNIP3L/NIX-mediated mitophagy plays a role in regulating mitochondrial ROS (mtROS) levels and preventing ferroptosis 88. BNIP3L/NIX releases Beclin-1 to induce mitophagy, and the released Beclin-1 binds to solute carrier family7 member 11 (SLC7A11), thereby inhibiting the cystine glutamate antiporter system Xc⁻, reducing cystine uptake and glutathione (GSH) synthesis, and consequently promoting ferroptosis through GSH depletion, GPX4 inactivation, and accumulation of ROS and lipid peroxides 80,89. Through the stimulation of the P62-KEAP1-NRF2 pathway, BNIP3-mediated mitophagy prevents ferroptosis and preserves iron and redox balance 90. The mitophagy receptor FUNDC1 has also been implicated in ferroptosis. FUNDC1 induces ferroptosis by binding to GPX4, facilitating its own mitochondrial transport via the TOM/TIM complex, and promoting the mitophagy dependent degradation of GPX4 91. FUNDC1 can also regulate ferroptosis via Acyl-CoA synthetase long-chain family member 4 (ACSL4) although the direction of this regulation requires further investigation 92. It should be noted that these receptor specific mechanisms have been characterized largely in nonmammary models, and their relevance to breast cancer remains to be determined. Direct evidence for mitophagy and ferroptosis crosstalk in breast cancer is now beginning to emerge. Xanthatin targets CDGSH iron sulfur domain 1 (CISD1) to trigger ferroptosis and PINK1/Parkin-dependent mitophagy in TNBC, establishing a dual anticancer mechanism 93. Platycodin D2 induces mitochondrial reactive oxygen species dependent ferroptosis and inhibits autophagic flux in breast cancer cells, with the two processes mutually reinforcing each other 94. In addition, LOX inhibition disrupts mitochondrial homeostasis and activates PINK1/Parkin-dependent mitophagy, generating ferroptotic vulnerability in TNBC 74.

VDACs are important proteins involved in mitophagy and ferroptosis. VDACs are targets of Parkin-mediated ubiquitination and act as mitochondrial docking sites, recruiting Parkin from the cytoplasm to damaged mitochondria and inducing mitophagy 95. VDACs allow the movement of iron and metabolites across the outer membrane, and erastin induced opening of VDACs accelerates ferroptosis 96. Furthermore, the redox-sensitive 2Fe-2S cluster protein, known as the OMM protein CISD1, is crucial for both iron uptake and regulation, as well as for maintaining ROS homeostasis. CISD1 oxidizes VDACs, leading to pore closure and impaired transport of mitochondrial metabolites 97. CISD1 mediated VDAC oxidation blocks mitochondrial TCA cycling, causing electron leakage from the electron transport chain and increased mitochondrial ROS production 98,99. The excess mitochondrial ROS then promotes extramitochondrial lipid peroxidation, ultimately contributing to ferroptosis 100,101.

Inhibition of mitophagy promotes or prevents ferroptosis. By increasing mitochondrial NAD⁺ via complex I upregulation, DMT1 deficiency promotes SIRT3-mediated deacetylation and activation of IDH2, leading to enhanced GSH-dependent antioxidant defense and ferroptosis suppression, as reported by Tan et al 102. FMS-like tyrosine kinase 3 (FLT3) overexpression inhibits autophagy and ferroptosis in breast cancer cells, and the addition of mitophagy inducers restores this regulation. In addition, the transcription factor AP-2γ mediates FLT3 transcriptional activation, further inhibiting mitophagy-induced ferroptosis 103. Accumulating evidence suggests that tumors with defective mitophagy are hypersensitive to ferroptosis induction, a phenomenon mechanistically linked to the unrestrained accumulation of mitochondrial ROS that relieves the suppression of lipid peroxidation 104. Figure 3 provides a schematic overview of the molecular interplay between ferroptosis and mitophagy.

## 6. Mitophagy in Breast Cancer Therapy

As a crucial mechanism for the selective elimination of damaged mitochondria, mitophagy contributes significantly to the maintenance of cellular homeostasis. However, in cancer, tumor cells frequently "take over" mitophagy to promote therapeutic resistance, metabolic adaptability, and survival. Targeting mitophagy has become a novel cancer treatment approach. Mitophagy exerts dual effects in cancer treatment. While it suppresses tumor growth by inducing metabolic dysfunction and DNA damage through mitochondrial destruction, it conversely fosters treatment resistance by enhancing tumor cell survival through the removal of therapy-damaged mitochondria 105. Table 2 summarizes pharmacological agents that have been reported to modulate mitophagy in breast cancer.

### 6.1 Overactivation of mitophagy in breast cancer

The overactivation of mitophagy in breast cancer leads to the destruction of mitochondria and the depletion of energy stores. The activation of mitophagy may facilitate the clearance of damaged mitochondria, consequently inhibiting the viability and proliferation of tumor cells. Many drugs can treat breast cancer by directly activating mitophagy, for example, it was found that Cyclovirobuxine D induces mitophagy through activation of the FOXO3a/PINK1-Parkin pathway, thereby inhibiting the development of triple-negative breast cancer 106. By altering the potential of the mitochondrial membrane and encouraging cellular autophagy through the AMPK/mTOR pathway, idebenone further suppresses the growth of TNBC cells 107. In TNBC, Guangsangon E causes mitochondrial dysfunction, mitophagy, and non-apoptotic cell death 108. Polyphyllin I increases mitochondrial PINK1 levels and induces mitophagic and apoptotic cell death in breast cancer 109. In addition, Gramicidin A, an exogenous ionophoric peptide, accumulates in mitochondria, decreases ATP levels, and induces mitophagy, thereby inhibiting the growth of MCF-7 cells 110.

### 6.2 Improving drug resistance in breast cancer through mitophagy

Senescent cells are more susceptible to the mitochondrial effects of fisetin derivatives than proliferating cells in breast cancer, an effect that leads to the removal of drug-resistant senescent breast cancer cells. This is in contrast to mitochondrial fisetin-induced cytotoxicity, which is linked to elevated levels of phosphorylated AMPK, decreased levels of AKT and HSP90, and impaired mitophagy 111. The Pep-1-mitochondrial membrane complex potentiates doxorubicin sensitivity in breast cancer by inducing mitochondrial hyperfusion and mitophagy 112. The miR-218-5p/ doxorubicin combination suppresses mitochondrial fusion in breast cancer cells, through the inhibition of Parkin-dependent mitophagy, leading to enhanced anticancer efficacy 113. Programmed death ligand 1 (PD-L1) expression is independently associated with high-risk clinicopathological features, contributing to a worse prognosis in primary breast cancer patients 114. Consequently, a crucial part of cancer treatment is immune checkpoint blockade therapy that targets PD-1/PD-L1 115. Although initially reported in colorectal cancer, Shen Qi Yi Chang (SQYC), a traditional Chinese medicine formula for colorectal cancer treatment, enhances PD-1 blockade via PINK1-Parkin-mediated dendritic cell mitophagy 116. Additionally, mitochondrial redirection of PD-L1, focused on the ATAD3a-PINK1 axis, provides an effective strategy for reversing chemotherapy resistance in breast cancer 117.

### 6.3 Targeting novel targets associated with mitophagy in breast cancer

Multiple molecular targets associated with the mitophagy pathway are being increasingly investigated, and breast cancer therapy can also be achieved by modulating these targets. For example, mesencephalic astrocyte-derived neurotrophic factor (MANF) promotes breast cancer cell survival under glucose starvation conditions through PRKN-mediated regulation of mitophagy 118. Early B cell factor 1 (EBF1), a key regulator of TNBC mitochondrial homeostasis, prevents widespread cell death induced by mitophagy by controlling the activity of HIF1α 119. Mitochondrial uncoupling protein 1 (UCP1) inhibits TNBC progression through activation of mitophagy 120. Accordingly, MANF, EBF1, and UCP1 constitute potential mitochondrial targets for therapeutic intervention in breast cancer.

### 6.4 Delivery systems associated with mitophagy in the treatment of breast cancer

Nanotechnology has become a viable strategy to enhance drug delivery and therapeutic results in addition to conventional targeted therapies. By encasing medications in nanoparticles, nanodelivery devices enable regulated drug release in vivo and lessen the impact of comorbidities 121,122. The combination of hydrogel-based chimeric antigen receptor T cell (CAR-T) cell delivery and BC1618 injection improved outcomes in triple-negative breast cancer by inducing a local inflammatory microenvironment and enhanced mitophagy that promoted CAR-T cell proliferation for sustained anti-tumor activity 123. There is also an all-in-one tumor therapy strategy that combines non-invasive sonodynamic therapy (SDT) enhanced by nanosensitizers with mitophagy inhibition. This approach combines SDT with mitophagy inhibition, thereby blocking the clearance of damaged mitochondria and ultimately inducing cell death through mitochondrial dysfunction and impaired autophagic flux 124. Synergizing cannabidiol with carbon monoxide nanocomplexes enhances TNBC therapy through excessive autophagy 125. TPP-SS-ATS-LS, a mitochondria-targeted artesunate smart prodrug liposome, targeting both tumor cells and mitochondria, acts by regulating PHB2 and PINK1 expression to inhibit breast cancer cell proliferation through mitophagy 126. Beyond the delivery systems described above, several other pharmacological modulators of mitophagy in breast cancer have been reported, including chlorpromazine and luteolin 127,128. A more comprehensive list of these agents is provided in Table 2 127-134.

### 6.5 Non-pharmaceutical therapies in the treatment of breast cancer through mitophagy

Strategies that utilize non-pharmacological therapies to promote or inhibit mitophagy for the treatment of breast cancer are also beginning to emerge. In a mouse model, high-intensity interval training emerged as an effective intervention against breast cancer, reducing tumor burden through the maintenance of mitophagy, which highlights its promise as a novel therapeutic approach 135. It has also been shown that fasting can enhance the efficacy of sorafenib in breast cancer through ROS-induced p53 pathway-mediated mitophagy 136. While animal and cellular studies have validated mitophagy-targeting agents against breast cancer and outlined their mechanisms, clinical trials are still absent. This gap underscores the critical next step: translating these promising preclinical results into viable clinical therapies.

## 7. Conclusions and Perspectives

This review outlines the core mechanisms of the mitophagy pathway, as well as the expression patterns and roles of molecules linked to mitophagy in breast cancer. A thorough understanding of these molecules may aid in identifying new therapeutic targets for breast cancer, and may also facilitate the detection of mitophagy activity within tumors. In order to shed light on the function of mitophagy in the development of breast cancer, we also summarized the impact of this process on the growth and spread of breast tumors. A key challenge remains in translating mitophagy mechanisms into effective breast cancer treatments. The net effect of mitophagy, whether it suppresses or promotes tumorigenesis, is highly context-dependent, varying according to disease stage, molecular subtype, and the tumor microenvironment. The anti-tumor activity of a number of clinically employed anticancer agents involves the induction of various modes of RCD 8. Currently, many studies have also found that certain drugs can be used to treat breast cancer by inducing mitophagy 118,137. However, it is worth noting that the precise interaction between mitophagy and RCD needs further exploration. Therefore, a comprehensive and in-depth understanding of the effects of mitophagy is required to formulate effective treatment strategies for breast cancer.

Future research may prioritize several key directions. Firstly, developing subtype-specific biomarkers and mitophagy inhibitors is crucial. Subsequently, nanotechnology could enable tumor-targeted drug delivery. It is also essential to investigate the dynamic crosstalk between mitophagy and the tumor microenvironment. Finally, exploring synergies between non-pharmacological and pharmaceutical interventions could minimize side effects and improve patient survival.

## Author Contributions

XY and LZ collected literature and wrote the manuscript; XY and YTY drew the figures and tables; LZ and SYC conceived the topic area and supervised; CYW and PL reviewed and edited the manuscript drafts. All authors commented on previous versions of the manuscript. All authors read and approved the final manuscript.

## Figures and Tables

**Figure 1:**
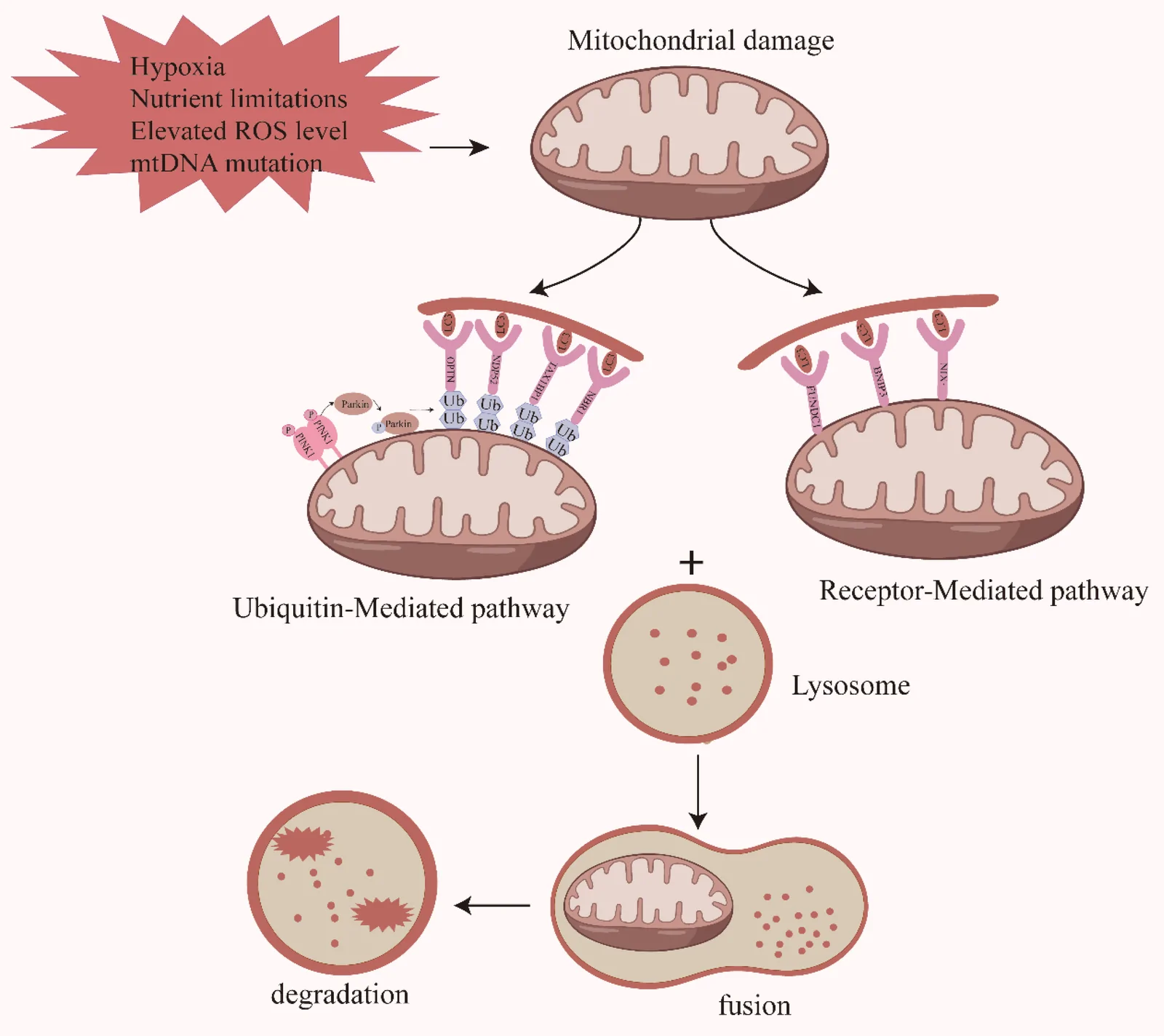
** Two mechanisms of mitophagy.** When mitochondria are damaged by external stimuli, two distinct pathways are activated to remove the damaged organelles. On the one hand, PINK1 is stabilized on OMM, where it phosphorylates ubiquitin at Ser65 and Parkin at Ser65, leading to Parkin activation and polyubiquitin chain formation on OMM proteins. Parkin-coupled polyubiquitin chains recruit autophagy adaptor proteins, such as OPTN, NDP52, TAX1BP1, and NBR1, which serve as molecular bridges that deliver the ubiquitinated mitochondrion to the autophagosome, where it subsequently fuses with the lysosome for degradation. On the other hand, the ubiquitin-independent mitophagy pathway mainly relies on mitophagy receptor proteins, such as BNIP3L/NIX, BNIP3, and FUNDC1, which directly interact with LC3 to initiate mitophagy without ubiquitination, followed by autophagosome-lysosome fusion and degradation of the damaged mitochondria.

**Figure 2:**
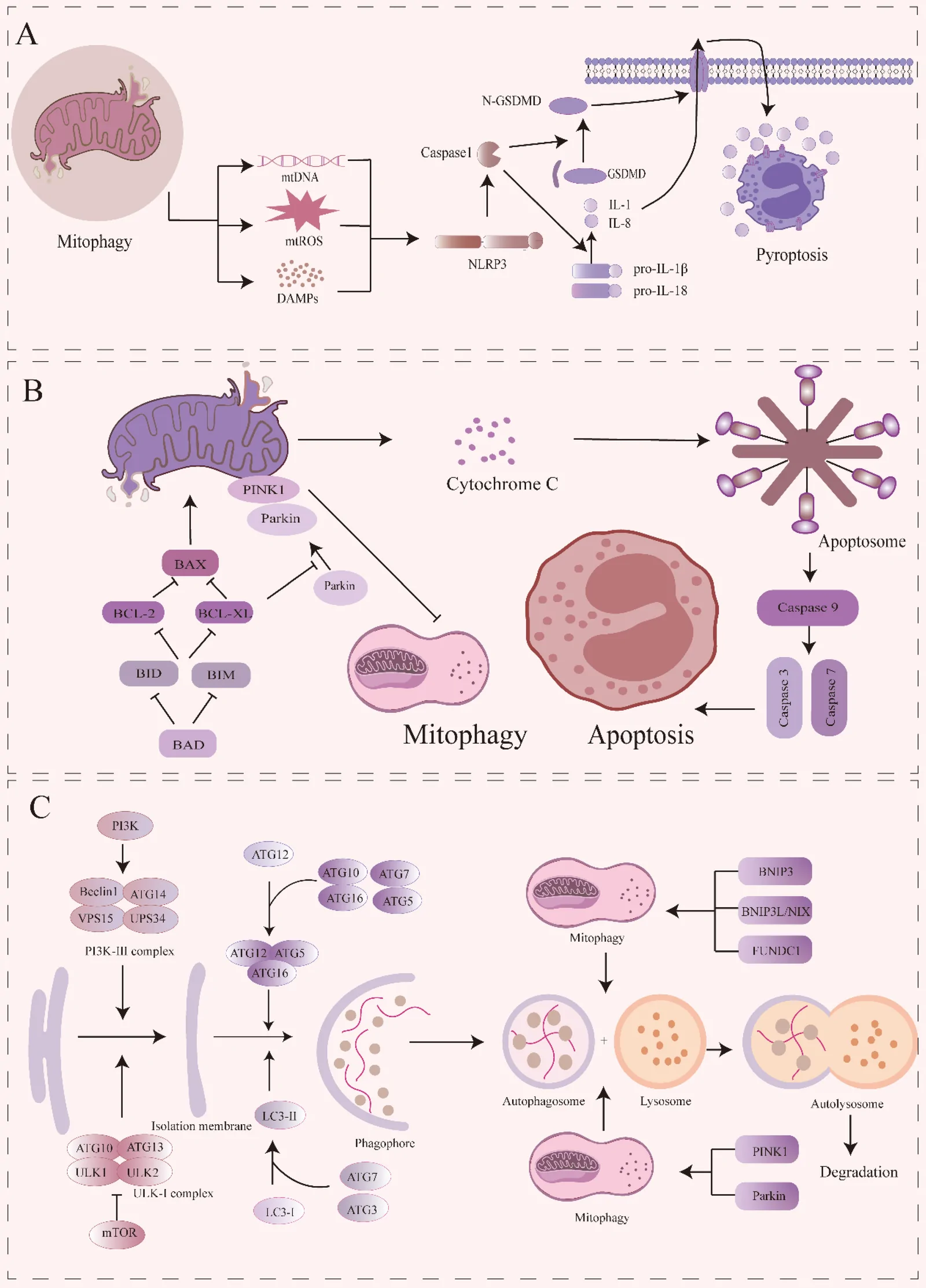
** Molecular crosstalk between mitophagy and cell death subroutines. A. Pyroptosis.** Damage-associated molecular patterns (DAMPs) and mitochondrial DNA (mtDNA) released from damaged mitochondria promote the formation of NLRP3 inflammasomes and trigger caspase-1. Gasdermin D (GSDMD) is cleaved by active caspase-1, which simultaneously cleaves pro-interleukin-1β (pro-IL-1β) and pro-IL-18 into their mature forms. Pyroptosis results from the oligomerization of the N-terminal fragment of GSDMD, which creates a pore in the plasma membrane. Mitophagy limits NLRP3 inflammasome activation by removing damaged mitochondria and reducing mtDNA release. **B. Apoptosis.** Mitochondrial outer membrane permeabilization (MOMP) occurs in severely damaged mitochondria and promotes apoptosis by releasing cytochrome C, which triggers apoptosome formation. MOMP is regulated by the B-cell lymphoma 2 (BCL-2) family of proteins, which includes the anti-apoptotic BCL-2 and B-cell lymphoma-extra large (BCL-xL), and the pro-apoptotic BCL-2-associated X protein (BAX) and BCL-2 antagonist/killer (BAK). BCL-xL inhibits Parkin recruitment to the outer mitochondrial membrane, thereby inhibiting mitophagy. **C. Core autophagy/mitophagy machinery.** Autophagy is initiated by phagophore formation, which sequesters cytoplasmic components for degradation. The mechanistic target of rapamycin (mTOR) pathway controls the ULK1 complex, which is essential for this process. Phagophores expand and mature into autophagosomes, which fuse with lysosomes to form autolysosomes for degradation. The ATG family, Beclin-1, and LC3 coordinate vesicle nucleation, elongation, and autophagosome maturation. Mitophagy is a selective form of autophagy in which damaged mitochondria are engulfed by autophagosomes. BID: BH3-interacting domain death agonist; BAD: BCL-2-associated agonist of cell death; BIM: BCL-2-interacting mediator of cell death; PI3K: phosphoinositide 3-kinase; VPS34: vacuolar protein sorting 34.

**Figure 3:**
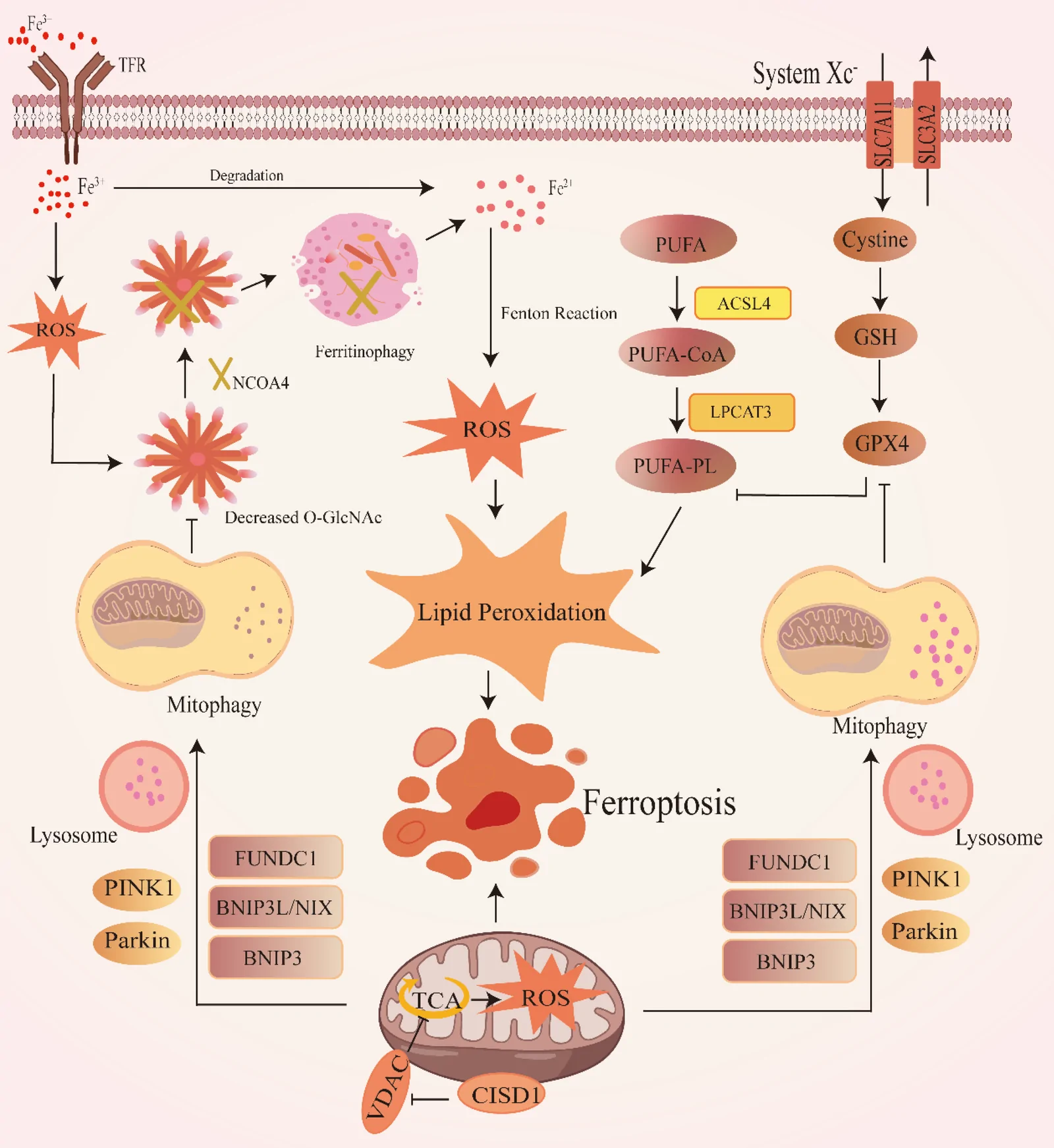
** Mitophagy interacts with ferroptosis.** Iron enters the cell via transferrin-mediated uptake through the transferrin receptor (TFR) on the cell membrane. Within the cell, Fe³⁺ is reduced to Fe²⁺ and stored in the labile iron pool, while excess iron is sequestered in ferritin. Inactivation of O-GlcNAc transferase reduces ferritin O-GlcNAcylation and activates NCOA4-mediated ferritinophagy, promoting ferritin degradation and increasing free iron levels. Free iron generates ROS via the Fenton reaction, leading to lipid peroxidation. ACSL4 catalyzes the conversion of polyunsaturated fatty acids (PUFAs) into PUFA-CoA, which is subsequently esterified into phospholipids by LPCAT3 to form PUFA-containing phospholipids (PUFA-PL). By promoting lipid peroxidation, LPCAT3 and ACSL4 are key mediators of ferroptosis. When the cystine/glutamate antiporter SLC7A11 (system Xc⁻) is blocked, cystine uptake decreases and intracellular cysteine levels fall, leading to decreased GSH synthesis and reduced GPX4 activity. The depletion of GPX4 and GSH results in the accumulation of ROS and lipid peroxides, leading to uncontrolled oxidative stress and ferroptosis. In addition, CISD1 oxidizes VDAC in a redox-dependent manner, leading to pore closure and impaired transport of mitochondrial metabolites. CISD1 mediated VDAC oxidation blocks mitochondrial TCA cycling, causing electron leakage from the electron transport chain and increased mitochondrial ROS production. The excess mitochondrial ROS then promotes extramitochondrial lipid peroxidation, ultimately contributing to ferroptosis. mtROS and iron overload subsequently trigger mitophagy through PINK1, Parkin, BNIP3, BNIP3L/NIX, and FUNDC1, which in turn modulates ferroptosis sensitivity depending on the receptor and cellular context.

**Table 1:** A brief comparison of two pathways of mitophagy

	Ubiquitin-dependent pathways	Receptor-mediated pathways
Main mechanisms	Ubiquitination of OMM proteins by PINK1-Parkin serves as a recognition signal for selective autophagy receptors, mediating autophagosomal engulfment and lysosomal degradation of the entire organelle, rather than clearance by the 26S proteasome	Direct binding to LC3 via mitochondrial outer membrane receptor proteins, independent of ubiquitination labeling
Key proteins/receptors	PINK1-Parkin, OPTN, NDP52, TAX1BP1, NBR1, p62	BNIP3, BNIP3L/NIX, FUNDC1
Ubiquitination	Parkin ubiquitinates OMM proteins including VDAC, MFN1, and MFN2, and recruits autophagy receptors; MUL1 restrains this process by maintaining ER-mitochondrial contacts	Independent of ubiquitination labeling; the receptor directly binds LC3 via its LIR motif
Common points	1. Both remove damaged mitochondria through autophagosomal engulfment and lysosomal degradation to maintain mitochondrial homeostasis 2. Dependent on LC3/GABARAP family of proteins, binding to autophagosome through LIR motifs 3. Ultimately degrade mitochondria through autolysosomes 4. Triggered by mitochondrial damage, oxidative stress, drug damage, and energy stress	

**Table 2:** Pharmacological modulators of mitophagy in breast cancer

Reagents	Type	Mitophagy related pathway	Effect on mitophagy	Reference
Cyclovirobuxine D	Natural compound	FOXO3a/PINK1-Parkin	Activates mitophagy	106
Idebenone	Synthetic compound	AMPK/mTOR	Activates autophagy/mitophagy	107
Guangsangon E	Natural compound	Mitochondrial dysfunction mediated mitophagy	Activates mitophagy	108
Polyphyllin I	Natural compound	PINK1	Activates mitophagy	109
Chlorpromazine	Repurposed drug	PINK1-Parkin	Activates mitophagy	127
Luteolin	Natural compound	SGK1-FOXO3a-BNIP3	Activates mitophagy	128
Flubendazole	Repurposed drug	EVA1A-DRP1	Activates mitophagy	129
Kaempferol	Natural compound	LKB1/AMPK/MFF	Activates mitophagy	130
Urolithin A	Natural compound	TFEB mediated mitophagy	Activates mitophagy	131
Baicalein	Natural compound	Autophagy mediated CDK1 downregulation	Sensitizes to doxorubicin	132
Liansinine	Natural compound	DNM1L mediated mitochondrial fission	Inhibits autophagy/mitophagy	133
Mitochondria-targeted ruthenium complexes	Synthetic compound	Autophagy inhibition	Inhibits autophagy	134

## Abbreviations

RCD: regulated cell death
ER-autophagy: endoplasmic reticulum autophagy
PINK1-Parkin: PTEN-induced putative kinase 1-Parkin RBR E3 ubiquitin-protein ligase
OMM: outer mitochondrial membrane
MUL1: mitochondrial E3 ubiquitin protein ligase 1
VDACs: voltage-dependent anion channels
MFN1 and MFN2: mitofusins
OPTN: optineurin
NDP52: nuclear dot protein 52 kDa/CALCOCO2
SQSTM1/p62: sequestosome 1
TAX1BP1: Tax1 binding protein 1
NBR1: neighbor of BRCA1 gene 1
UBDs: ubiquitin-binding domains
LIR: LC3-interacting region
ATG8: autophagy-related protein 8
LC3: microtubule-associated protein 1A/1B-light chain 3
GABARAP: GABA receptor-associated protein
BNIP3: BCL2/adenovirus E1B 19-kDa interacting protein 3
BNIP3L/NIX: BCL2 interacting protein 3 like/NIP3-like protein X
TM: single-channel C-terminal transmembrane
FUNDC1: FUN14 domain containing 1
CK2: casein kinase 2
BRCA1: breast cancer susceptibility gene 1
ATP: adenosine triphosphate
TCA: tricarboxylic acid cycle
ROS: reactive oxygen species
HIF1α: hypoxia-inducible factor 1 subunit alpha
TNBC: triple negative breast cancer
DMT1: divalent metal transporter 1
ULK1: Unc-51 like autophagy activating kinase 1
NLRP3: NLR family pyrin domain containing 3
OXPHOS: oxidative phosphorylation
LOX: Lysyl oxidase
NCOA4: nuclear receptor coactivator 4
O-GlcNAc: O-linked β-N-acetylglucosamine
SLC7A11: solute carrier family 7 member 11
GSH: glutathione
GPX4: glutathione peroxidase 4
ACSL4: acyl-CoA synthetase long-chain family member 4
CISD1: CDGSH iron sulfur domain 1
FLT3: FMS-like tyrosine kinase 3
PD-L1: programmed death ligand 1
SQYC: Shen Qi Yi Chang
MANF: mesencephalic astrocyte-derived neurotrophic factor
EBF1: early B cell factor 1
UCP1: uncoupling protein 1
CAR-T: chimeric antigen receptor T cell
SDT: sonodynamic therapy
DAMPs: damage-associated molecular patterns
mtDNA: mitochondrial DNA
GSDMD: gasdermin D
pro-IL-1β: pro-interleukin-1β
MOMP: mitochondrial outer membrane permeabilization
BCL-2: B-cell lymphoma 2
BCL-xL: B-cell lymphoma-extra large
BAX: BCL-2-associated X protein
BAK: BCL-2 antagonist/killer
mTOR: mechanistic target of rapamycin
BID: BH3-interacting domain death agonist
BAD: BCL-2-associated agonist of cell death
BIM: BCL-2-interacting mediator of cell death
PI3K: phosphoinositide 3-kinase
VPS34: vacuolar protein sorting 34
TFR: transferrin receptor
PUFAs: polyunsaturated fatty acids
PUFA-PL: PUFA-containing phospholipids
mtROS: mitochondrial ROS

## References

[1] Nierengarten MB (2025). Key findings highlighted in the first annual cancer report from the American College of Surgeons. Cancer.

[2] Giaquinto AN, Sung H, Newman LA (2024). Breast cancer statistics 2024. CA Cancer J Clin.

[3] Tian H, Ma D, Tan X (2021). Platinum and taxane based adjuvant and neoadjuvant chemotherapy in early triple-negative breast cancer: a narrative review. Front Pharmacol.

[4] Siddiqui SN, Haider MF, Rahman MA (2025). Innovative approaches in breast cancer therapy: repurposing nanocarriers for enhanced outcomes. Naunyn Schmiedebergs Arch Pharmacol.

[5] Tu S-N, Hu F, Zhang J (2025). Research progress on the signaling pathway mechanism of terpenoids against breast cancer. Discov Oncol.

[6] O'Shaughnessy J, Tolaney SM, Yardley DA (2025). Real-world risk of recurrence and treatment outcomes with adjuvant endocrine therapy in patients with stage II-III HR+/HER2- early breast cancer. Breast.

[7] Zhou S, Liu J, Wan A (2024). Epigenetic regulation of diverse cell death modalities in cancer: a focus on pyroptosis, ferroptosis, cuproptosis, and disulfidptosis. J Hematol Oncol.

[8] Liao M, Qin R, Huang W (2022). Targeting regulated cell death (RCD) with small-molecule compounds in triple-negative breast cancer: a revisited perspective from molecular mechanisms to targeted therapies. J Hematol Oncol.

[9] Chen D, Chen X, Yang M (2025). H3K36me2 methyltransferase NSD2/WHSC1 promotes triple-negative breast cancer metastasis via activation of ULK1-dependent autophagy. Autophagy.

[10] Liu D, Qin H, Gao Y (2024). Cardiovascular disease: Mitochondrial dynamics and mitophagy crosstalk mechanisms with novel programmed cell death and macrophage polarisation. Pharmacol Res.

[11] Li J, Yang D, Li Z (2023). PINK1/Parkin-mediated mitophagy in neurodegenerative diseases. Ageing Res Rev.

[12] Zhu L, Xu Y, Lei J (2024). Molecular mechanism and potential role of mitophagy in acute pancreatitis. Mol Med.

[13] Wang S, Long H, Hou L (2023). The mitophagy pathway and its implications in human diseases. Signal Transduct Target Ther.

[14] Guo ZJ, Yu Q, Sha R (2026). PINK1-mediated mitophagy enhances breast cancer proliferation through metabolic reprogramming. Oncol Rep.

[15] Liu Y, Wang H, Zhang S (2025). The role of mitochondrial biogenesis, mitochondrial dynamics and mitophagy in gastrointestinal tumors. Cancer Cell Int.

[16] Tiwari SK, Chandrasekharan A, Lupitha SS (2025). Hypoxia induced mitophagy generates reversible metabolic and redox heterogeneity with transient cell death switch driving tumorigenesis. Free Radic Biol Med.

[17] Hu Y, Li Y, Li M (2024). Calcium supplementation attenuates fluoride-induced bone injury via PINK1/Parkin-mediated mitophagy and mitochondrial apoptosis in mice. J Hazard Mater.

[18] Gonçalves FB, Morais VA (2021). PINK1: A bridge between mitochondria and parkinson's disease. Life.

[19] Koyano F, Okatsu K, Kosako H (2014). Ubiquitin is phosphorylated by PINK1 to activate parkin. Nature.

[20] Kane LA, Lazarou M, Fogel AI (2014). PINK1 phosphorylates ubiquitin to activate Parkin E3 ubiquitin ligase activity. J Cell Biol.

[21] Kazlauskaite A, Kondapalli C, Gourlay R (2014). Parkin is activated by PINK1-dependent phosphorylation of ubiquitin at Ser65. Biochem J.

[22] Ordureau A, Sarraf SA, Duda DM (2014). Quantitative proteomics reveal a feedforward mechanism for mitochondrial PARKIN translocation and ubiquitin chain synthesis. Mol Cell.

[23] Kazlauskaite A, Martínez-Torres RJ, Wilkie S (2015). Binding to serine 65-phosphorylated ubiquitin primes Parkin for optimal PINK1-dependent phosphorylation and activation. EMBO Rep.

[24] Orvedahl A, Sumpter R Jr, Xiao G (2011). Image-based genome-wide siRNA screen identifies selective autophagy factors. Nature.

[25] Mukherjee R, Chakrabarti O (2016). Ubiquitin-mediated regulation of the E3 ligase GP78 by MGRN1 in trans affects mitochondrial homeostasis. J Cell Sci.

[26] Szargel R, Shani V, Abd Elghani F (2016). The PINK1, synphilin-1 and SIAH-1 complex constitutes a novel mitophagy pathway. Hum Mol Genet.

[27] Liu H, Deng Y, Peng M (2025). ARIH1 promotes preeclampsia by inducing MFN2-dependent hypoxia-triggered mitophagy and endoplasmic reticulum stress in trophoblasts. FASEB J.

[28] Kumar A, Zacharioudakis E (2026). Mitochondrial ubiquitination as a signaling hub: balancing mitophagy, inflammation, and cell death. Biochemistry.

[29] Geisler S, Holmström KM, Skujat D (2010). PINK1/Parkin-mediated mitophagy is dependent on VDAC1 and p62/SQSTM1. Nat Cell Biol.

[30] Rakovic A, Grünewald A, Kottwitz J (2011). Mutations in PINK1 and Parkin impair ubiquitination of Mitofusins in human fibroblasts. PLoS One.

[31] Puri R, Cheng XT, Lin MY (2019). Mul1 restrains Parkin-mediated mitophagy in mature neurons by maintaining ER-mitochondrial contacts. Nat Commun.

[32] Wong YC, Holzbaur EL (2014). Optineurin is an autophagy receptor for damaged mitochondria in parkin-mediated mitophagy that is disrupted by an ALS-linked mutation. Proc Natl Acad Sci USA.

[33] Verlhac P, Grégoire IP, Azocar O (2015). Autophagy receptor NDP52 regulates pathogen-containing autophagosome maturation. Cell Host Microbe.

[34] Bauer B, Idinger J, Schuschnig M, Ferrari L, Martens S (2024). Recruitment of autophagy initiator TAX1BP1 advances aggrephagy from cargo collection to sequestration. EMBO J.

[35] Kirkin V, Lamark T, Sou YS (2009). A role for NBR1 in autophagosomal degradation of ubiquitinated substrates. Mol Cell.

[36] Johansen T, Lamark T (2020). Selective autophagy: ATG8 family proteins, LIR motifs and cargo receptors. J Mol Biol.

[37] Wang W, Liu J, Li J (2025). A PRKN-independent mechanism regulating cardiac mitochondrial quality control. Autophagy.

[38] Padman BS, Nguyen TN, Uoselis L (2019). LC3/GABARAPs drive ubiquitin-independent recruitment of Optineurin and NDP52 to amplify mitophagy. Nat Commun.

[39] Onishi M, Yamano K, Sato M (2021). Molecular mechanisms and physiological functions of mitophagy. EMBO J.

[40] Zhu Y, Massen S, Terenzio M (2013). Modulation of serines 17 and 24 in the LC3-interacting region of Bnip3 determines pro-survival mitophagy versus apoptosis. J Biol Chem.

[41] Marinković M, Novak I (2021). A brief overview of BNIP3L/NIX receptor-mediated mitophagy. FEBS Open Bio.

[42] Marinković M, Šprung M, Novak I (2021). Dimerization of mitophagy receptor BNIP3L/NIX is essential for recruitment of autophagic machinery. Autophagy.

[43] Nguyen-Dien GT, Kozul K, Cui Y (2023). FBXL4 suppresses mitophagy by restricting the accumulation of NIX and BNIP3 mitophagy receptors. EMBO J.

[44] He YL, Li J, Gong SH (2022). BNIP3 phosphorylation by JNK1/2 promotes mitophagy via enhancing its stability under hypoxia. Cell Death Dis.

[45] Liu L, Feng D, Chen G (2012). Mitochondrial outer-membrane protein FUNDC1 mediates hypoxia-induced mitophagy in mammalian cells. Nat Cell Biol.

[46] Pan P, Chen J, Liu X (2021). FUNDC1 regulates autophagy by inhibiting ROS-NLRP3 signaling to avoid apoptosis in the lung in a lipopolysaccharide-induced mouse model. Shock.

[47] Liu R, Xu C, Zhang W (2022). FUNDC1-mediated mitophagy and HIF1α activation drives pulmonary hypertension during hypoxia. Cell Death Dis.

[48] Li K, Xia X, Tong Y (2024). Multiple roles of mitochondrial autophagy receptor FUNDC1 in mitochondrial events and kidney disease. Front Cell Dev Biol.

[49] Lv M, Wang C, Li F (2017). Structural insights into the recognition of phosphorylated FUNDC1 by LC3B in mitophagy. Protein Cell.

[50] Kuang Y, Ma K, Zhou C (2016). Structural basis for the phosphorylation of FUNDC1 LIR as a molecular switch of mitophagy. Autophagy.

[51] Wang J, Zhu P, Li R (2019). Fundc1-dependent mitophagy is obligatory to ischemic preconditioning-conferred renoprotection in ischemic AKI via suppression of Drp1-mediated mitochondrial fission. Redox Biol.

[52] Tang T, Hu L, Ding C (2023). Src inhibition rescues FUNDC1-mediated neuronal mitophagy in ischaemic stroke. Stroke Vasc Neurol.

[53] Zhu L, Wu W, Jiang S (2020). Pan-cancer analysis of the mitophagy-related protein PINK1 as a biomarker for the immunological and prognostic role. Front Oncol.

[54] Miyahara K, Takano N, Yamada Y (2021). BRCA1 degradation in response to mitochondrial damage in breast cancer cells. Sci Rep.

[55] Chida K, Oshi M, Roy AM (2024). Enhanced cancer cell proliferation and aggressive phenotype counterbalance in breast cancer with high BRCA1 gene expression. Breast Cancer Res Treat.

[56] Li J, Xu X, Huang H (2022). Pink1 promotes cell proliferation and affects glycolysis in breast cancer. Exp Biol Med (Maywood).

[57] Sun X, Ye G, Li J (2024). The tumor suppressor Parkin exerts anticancer effects through regulating mitochondrial GAPDH activity. Oncogene.

[58] Liu J, Zhang C, Wu H (2020). Parkin ubiquitinates phosphoglycerate dehydrogenase to suppress serine synthesis and tumor progression. J Clin Invest.

[59] Wahabi K, Perwez A, Kamarudheen S (2019). Parkin gene mutations are not common, but its epigenetic inactivation is a frequent event and predicts poor survival in advanced breast cancer patients. BMC Cancer.

[60] Tan N, Liu T, Wang X (2022). The multi-faced role of FUNDC1 in mitochondrial events and human diseases. Front Cell Dev Biol.

[61] Yuan Q, Sun N, Zheng J (2020). Prognostic and immunological role of FUN14 domain containing 1 in pan-cancer: friend or foe?. Front Oncol.

[62] Wu L, Zhang D, Zhou L (2019). FUN14 domain-containing 1 promotes breast cancer proliferation and migration by activating calcium-NFATC1-BMI1 axis. EBioMedicine.

[63] Yu Q, Fu W, Fu Y (2023). BNIP3 as a potential biomarker for the identification of prognosis and diagnosis in solid tumours. Mol Cancer.

[64] Mauro-Lizcano M, Sotgia F, Lisanti MP (2024). Mitophagy and cancer: role of BNIP3/BNIP3L as energetic drivers of stemness features, ATP production, proliferation, and cell migration. Aging (Albany NY).

[65] Degli Esposti M (2024). Did mitophagy follow the origin of mitochondria?. Autophagy.

[66] Ganley IG, Simonsen A (2022). Diversity of mitophagy pathways at a glance. J Cell Sci.

[67] Li L, Hu F (2023). Mitophagy in tumor: foe or friend?. Endokrynol Pol.

[68] Barra J, Crosbourne I, Roberge CL (2024). DMT1-dependent endosome-mitochondria interactions regulate mitochondrial iron translocation and metastatic outgrowth. Oncogene.

[69] Singh K, Roy M, Prajapati P (2019). NLRX1 regulates TNF-α-induced mitochondria-lysosomal crosstalk to maintain the invasive and metastatic potential of breast cancer cells. Biochim Biophys Acta Mol Basis Dis.

[70] Deng R, Zhang H-L, Huang J-H (2021). MAPK1/3 kinase-dependent ULK1 degradation attenuates mitophagy and promotes breast cancer bone metastasis. Autophagy.

[71] Li X, Wang M, Li S (2021). HIF-1-induced mitochondrial ribosome protein L52: a mechanism for breast cancer cellular adaptation and metastatic initiation in response to hypoxia. Theranostics.

[72] Chen Q, Lei JH, Bao J (2020). BRCA1 deficiency impairs mitophagy and promotes inflammasome activation and mammary tumor metastasis. Adv Sci (Weinh).

[73] Liu S, Liu C, Zheng W (2026). Epigenetic reactivation of TNFRSF19 suppresses mitophagy and sensitizes triple-negative breast cancer to doxorubicin. Adv Sci (Weinh).

[74] Saatci O, Ulukan B, Cetin M (2026). Lysyl oxidase inhibition disrupts mitochondrial homeostasis to create vulnerability to ferroptosis in TNBC. Cell Rep Med.

[75] Nie S, Liu D, Hu J (2026). TBX20 promotes doxorubicin resistance in breast cancer cells through enhanced ABCC1 expression and inhibition of mitophagy. PLoS One.

[76] Jin X, Liu Y, Zheng X (2026). CIDEC impairs mitochondrial fitness and blocks protective autophagy via the inhibition of cGMP/PKG pathway to exert tumor-suppressive effects in breast cancer. Cell Oncol (Dordr).

[77] Ye Y, Zeng Q, Ou Z (2026). Mitochondrial pyruvate dehydrogenase kinase 1 drives bevacizumab resistance and malignant phenotype of TNBC by enhancing mitophagy. Pharmacol Res.

[78] Li J, Cao F, Yin H (2020). Ferroptosis: past, present and future. Cell Death Dis.

[79] Wu H, Liu Q, Shan X (2023). ATM orchestrates ferritinophagy and ferroptosis by phosphorylating NCOA4. Autophagy.

[80] Song X, Zhu S, Chen P (2018). AMPK-Mediated BECN1 Phosphorylation Promotes Ferroptosis by Directly Blocking System X_c_^-^ Activity. Curr Biol.

[81] Ji H, Zhao Y, Ma X (2024). Upregulation of UHRF1 promotes PINK1-mediated mitophagy to alleviates ferroptosis in diabetic nephropathy. Inflammation.

[82] Lee S, Hwang N, Seok BG (2023). Autophagy mediates an amplification loop during ferroptosis. Cell Death Dis.

[83] Wu K, Zhao W, Hou Z (2025). Ferritinophagy: multifaceted roles and potential therapeutic strategies in liver diseases. Front Cell Dev Biol.

[84] Singh LP, Yumnamcha T, Devi TS (2021). Mitophagy, ferritinophagy and ferroptosis in retinal pigment epithelial cells under high glucose conditions: implications for diabetic retinopathy and age-related retinal diseases. JOJ Ophthalmol.

[85] Yu F, Zhang Q, Liu H (2022). Dynamic O-GlcNAcylation coordinates ferritinophagy and mitophagy to activate ferroptosis. Cell Discov.

[86] Chen H, Zhou X, Ma J (2024). Low-dose deoxynivalenol exposure triggers hepatic excessive ferritinophagy and mitophagy mitigated by hesperidin modulated O-GlcNAcylation. J Hazard Mater.

[87] Zhang L, Hou L, Liu Z (2020). A mitophagic response to iron overload-induced oxidative damage associated with the PINK1/Parkin pathway in pancreatic beta cells. J Trace Elem Med Biol.

[88] Yamashita SI, Sugiura Y, Matsuoka Y (2024). Mitophagy mediated by BNIP3 and NIX protects against ferroptosis by downregulating mitochondrial reactive oxygen species. Cell Death Differ.

[89] Eskander G, Abdelhamid, SG, Wahdan SA (2025). Insights on the crosstalk among different cell death mechanisms. Cell Death Discov.

[90] Wang XX, Li M, Xu XW (2025). BNIP3-mediated mitophagy attenuates hypoxic-ischemic brain damage in neonatal rats by inhibiting ferroptosis through P62-KEAP1-NRF2 pathway activation to maintain iron and redox homeostasis. Acta Pharmacol Sin.

[91] Bi Y, Liu S, Qin X (2024). FUNDC1 interacts with GPx4 to govern hepatic ferroptosis and fibrotic injury through a mitophagy-dependent manner. J Adv Res.

[92] Li FJ, Hu H, Wu L (2024). Ablation of mitophagy receptor FUNDC1 accentuates septic cardiomyopathy through ACSL4-dependent regulation of ferroptosis and mitochondrial integrity. Free Radic Biol Med.

[93] Liu Q, Chen H, Li X (2026). Xanthatin Targets CISD1 to drive ferroptosis and mitophagy as a dual anticancer strategy in triple-negative breast cancer. Adv Sci (Weinh).

[94] Li Y, Lin H, Sun Y (2025). Platycodin D2 mediates incomplete autophagy and ferroptosis in breast cancer cells by regulating mitochondrial ROS. Phytother Res.

[95] Zhang L, Hu Z, Li Z (2023). Crosstalk among mitophagy, pyroptosis, ferroptosis, and necroptosis in central nervous system injuries. Neural Regen Res.

[96] Wang W, Thomas ER, Xiao R (2025). Targeting mitochondria-regulated ferroptosis: A new frontier in Parkinson's Disease therapy. Neuropharmacology.

[97] Lemasters JJ (2017). Evolution of voltage-dependent anion channel function: from molecular sieve to governator to actuator of ferroptosis. Front Oncol.

[98] Fujii J, Imai H (2024). Oxidative metabolism as a cause of lipid peroxidation in the execution of ferroptosis. Int J Mol Sci.

[99] Lyamzaev KG, Huan H, Panteleeva AA (2024). Exogenous iron induces mitochondrial lipid peroxidation, lipofuscin accumulation, and ferroptosis in H9c2 cardiomyocytes. Biomolecules.

[100] Jiang Y, Liu X, Sun M (2025). The mist of ferroptosis: The orpheus journey of mitochondria - exploring the symphony of cell fate. Int J Biol Macromol.

[101] Merkel M, Goebel B, Boll M (2023). Mitochondrial reactive oxygen species formation determines ACSL4/LPCAT2-mediated ferroptosis. Antioxidants (Basel).

[102] Tan Q, Zhang X, Li S (2023). DMT1 differentially regulates mitochondrial complex activities to reduce glutathione loss and mitigate ferroptosis. Free Radic Biol Med.

[103] Shen J, He Y, Zhou B (2024). TFAP2C/FLT3 axis reduces ferroptosis in breast cancer cells by inhibiting mitochondrial autophagy. Int J Biochem Cell Biol.

[104] Liu S, Chen JH, Li LC (2025). Susceptibility of mitophagy-deficient tumors to ferroptosis induction by relieving the suppression of lipid peroxidation. Adv Sci (Weinh).

[105] Liu H, Song Y, Wang H (2025). Deciphering the power of resveratrol in mitophagy: from molecular mechanisms to therapeutic applications. Phytother Res.

[106] Wang ZQ, Wu ZX, Chen JW (2024). Cyclovirobuxine D inhibits triple-negative breast cancer via YAP/TAZ suppression and activation of the FOXO3a/PINK1-Parkin pathway-induced mitophagy. Phytomedicine.

[107] Zhang Y, Yang F, Wu J (2024). Idebenone Exerts anti-Triple Negative Breast Cancer Effects via Dual Signaling Pathways of GADD45 and AMPK. Nutr Cancer.

[108] Shen Y, Han Z, Wang L (2022). Guangsangon E triggers mitochondria dysfunction and mitophagy in triple-negative breast cancer and leads to non-apoptotic cell death. Mol Carcinog.

[109] Li GB, Fu RQ, Shen HM (2017). Polyphyllin I induces mitophagic and apoptotic cell death in human breast cancer cells by increasing mitochondrial PINK1 levels. Oncotarget.

[110] Xue YW, Itoh H, Dan S (2022). Gramicidin A accumulates in mitochondria, reduces ATP levels, induces mitophagy, and inhibits cancer cell growth. Chem Sci.

[111] Rzeszutek I, Cybularczyk-Cecotka M, Deręgowska A (2024). New mitochondria-targeted fisetin derivative compromises mitophagy and limits survival of drug-induced senescent breast cancer cells. J Med Chem.

[112] Chang JC, Chang HS, Yeh CY (2022). Regulation of mitochondrial fusion and mitophagy by intra-tumoral delivery of membrane-fused mitochondria or Midiv-1 enhances sensitivity to doxorubicin in triple-negative breast cancer. Biomed Pharmacother.

[113] Naso FD, Bruqi K, Manzini V (2024). miR-218-5p and doxorubicin combination enhances anticancer activity in breast cancer cells through Parkin-dependent mitophagy inhibition. Cell Death Discov.

[114] Soltani M, Abbaszadeh M, Fouladseresht H (2025). PD-L1 importance in malignancies comprehensive insights into the role of PD-L1 in malignancies: from molecular mechanisms to therapeutic opportunities. Clin Exp Med.

[115] Nie H, Peng L, Yang T (2025). 68Ga-Labeled Peptide for Noninvasive Quantifying Tumor Exposure of PD-L1 Therapeutics. ACS Omega.

[116] Wang H, Ji Y, Deng S (2025). SQYC formula improves the efficacy of PD-1 monoclonal antibodies in MSS colorectal cancer by regulating dendritic cell mitophagy via the PINK1-Parkin pathway. Phytomedicine.

[117] Xie XQ, Yang Y, Wang Q (2023). Targeting ATAD3A-PINK1-mitophagy axis overcomes chemoimmunotherapy resistance by redirecting PD-L1 to mitochondria. Cell Res.

[118] Xiong Z, Yang L, Zhang C (2025). MANF facilitates breast cancer cell survival under glucose-starvation conditions via PRKN-mediated mitophagy regulation. Autophagy.

[119] Qiu Z, Guo W, Dong B (2022). EBF1 promotes triple-negative breast cancer progression by surveillance of the HIF1α pathway. Proc Natl Acad Sci U S A.

[120] Xia J, Chu C, Li W (2022). Mitochondrial protein UCP1 inhibits the malignant behaviors of triple-negative breast cancer through activation of mitophagy and pyroptosis. Int J Biol Sci.

[121] Raj M, Meena A, Seth R (2025). An update on nanoformulations with FDA approved drugs for female reproductive cancer. J Microencapsul.

[122] Kong X, Xie X, Wu J (2025). Combating cancer immunotherapy resistance: a nano-medicine perspective. Cancer Commun (Lond).

[123] Li G, Du R, Wang D (2025). Improved efficacy of triple-negative breast cancer immunotherapy via hydrogel-based co-delivery of CAR-T cells and mitophagy agonist. Adv Sci (Weinh).

[124] Zheng Y, Zhang T, Chang M (2025). Sonoactivated z-scheme heterojunction for enhanced sonodynamic mitophagy inhibition and triple negative breast cancer treatment. Adv Mater.

[125] Xiao C, Sun Y, Fan J (2023). Engineering cannabidiol synergistic carbon monoxide nanocomplexes to enhance cancer therapy via excessive autophagy. Acta Pharm Sin B.

[126] Gu L, Zhang J, Liu D (2022). Development of artesunate intelligent prodrug liposomes based on mitochondrial targeting strategy. J Nanobiotechnology.

[127] Pu Y, Xu F, He A (2025). Repurposing chlorpromazine for the treatment of triple-negative breast cancer growth and metastasis based on modulation of mitochondria-mediated apoptosis and autophagy/mitophagy. Br J Cancer.

[128] Wu L, Lin Y, Gao S (2023). Luteolin inhibits triple-negative breast cancer by inducing apoptosis and autophagy through SGK1-FOXO3a-BNIP3 signaling. Front Pharmacol.

[129] Zhen Y, Yuan Z, Zhang J (2022). Flubendazole induces mitochondrial dysfunction and DRP1-mediated mitophagy by targeting EVA1A in breast cancer. Cell Death Dis.

[130] Cao J, Ma X, Yan X (2023). Kaempferol induces mitochondrial dysfunction and mitophagy by activating the LKB1/AMPK/MFF pathway in breast precancerous lesions. Phytother Res.

[131] Zheng B, Wang Y, Zhou B (2024). Urolithin A inhibits breast cancer progression via activating TFEB-mediated mitophagy in tumor macrophages. J Adv Res.

[132] Hua F, Xiao YY, Qu XH (2023). Baicalein sensitizes triple negative breast cancer MDA-MB-231 cells to doxorubicin via autophagy-mediated down-regulation of CDK1. Mol Cell Biochem.

[133] Zhou J, Li G, Zheng Y (2015). A novel autophagy/mitophagy inhibitor liensinine sensitizes breast cancer cells to chemotherapy through DNM1L-mediated mitochondrial fission. Autophagy.

[134] Zhang GD, Wang MM, Su Y (2024). Mitochondria-targeted ruthenium complexes can be generated in vitro and in living cells to target triple-negative breast cancer cells by autophagy inhibition. J Inorg Biochem.

[135] Khoramipour K, Soltany A, Khosravi P (2024). High intensity interval training as a therapy: Mitophagy restoration in breast cancer. Arch Biochem Biophys.

[136] Li R, Ma Y, He A (2025). Fasting enhances the efficacy of Sorafenib in breast cancer via mitophagy mediated ROS-driven p53 pathway. Free Radic Biol Med.

[137] Fang J, Zou X, Gong L (2023). Acid ground nano-realgar processed product inhibits breast cancer by inducing mitophagy via the p53/BNIP3/NIX pathway. J Cell Mol Med.

